# Granulocyte Colony-Stimulating Factor Combined With Transcutaneous Electrical Acupoint Stimulation in Treatment of Unresponsive Thin Endometrium in Frozen Embryo Transfer Cycles

**DOI:** 10.3389/frph.2021.647336

**Published:** 2021-07-08

**Authors:** Linjiang Song, Qinxiu Zhang, Shaomi Zhu, Xudong Shan

**Affiliations:** School of Medical and Life Sciences, Reproductive & Women-Children Hospital, Chengdu University of Traditional Chinese Medicine, Chengdu, China

**Keywords:** thin endometrium, FET, G-CSF, teas, embryo implantation rate

## Abstract

**Objective:** This trial was designed to assess the treatment effects of granulocyte colony-stimulating factor (G-CSF) and transcutaneous electrical acupoint stimulation (TEAS) on thin endometrium in frozen-thawed embryo transfer (FET) cycles.

**Methods:** Ninety-nine patients with previous cancellations of embryo transfer were included, 56 of whom were prospectively treated with intrauterine perfusion of G-CSF in subsequent FET cycles. The selected patients were randomized into the G-CSF perfusion only group and the G-CSF perfusion combined with TEAS group. The other 43 patients were retrospectively included as controls.

**Results:** Compared to previous cycles, endometrial thickness was statistically significantly increased in the two treatment groups (5.97 ± 0.60, 7.52 ± 0.56, 6.14 ± 0.52, and 7.66 ± 0.44; *P* = 0.00 and 0.00, respectively). The increases in endometrial thickness suggested that no statistically significant difference was found between the two treatment groups. The G-CSF with TEAS group suggested a higher embryo implantation rate than the G-CSF perfusion only and control groups (33.33 and 29.1% and 33.33 and 17.39%; *P* = 0.412 and 0.091, respectively). The G-CSF combined with TEAS group demonstrated nominally higher clinical and ongoing pregnancy rates than the G-CSF perfusion-only group and controls, though, the difference was not statistically significant.

**Conclusion:** G-CSF has a potential role in improving endometrium thickness in patients with thin unresponsive endometrium in FET treatment cycles. In addition, when combined with TEAS, G-CSF perfusion treatment also improves the embryo implantation rate; however, randomized controlled trials are highly demanded to provide high-grade evidence regarding clinical pregnancy rate after G-CSF perfusion treatment.

## Introduction

Endometrial thickness plays a major part in embryo implantation. Some studies have shown that adequate endometrium (more than 7 mm and preferably 9 mm) is a key precondition for successful implantation. Endometrium thickness below 7 mm is widely considered not preferable for embryo transfer and is associated with decreased pregnancy rates (1). Various therapies have been recommended such as low-dose aspirin, high-dose estrogen, transvaginal sildenafil citrate, and treatment with pentoxifylline and tocopherol (2). However, even after utilizing these remedies, many women are not able to achieve minimal endometrial thickness, and only limited options are advised: embryo transfers are canceled; embryos are cryopreserved, expecting better endometrium in subsequent cycles; or transfers are done regardless of the thin endometrium, accepting decreased chances for pregnancy. Therefore, effective therapeutic approaches for improving endometrial thickness in those women are urgently needed.

Granulocyte colony-stimulating factor (G-CSF) is one of the cytokine family members including colony-stimulating factors. It has been reported to have effects on embryo implantation and development and contribute to stromal cells decidualization and trophoblast cells invasion into the maternal tissue (3, 4). Studies have demonstrated a benefit from G-CSF perfusion treatment with repeated implantation failure, suggesting that G-CSF treatment has positive effects on the endometrium (5, 6). In addition, previous studies reported successful treatment with G-CSF perfusion in patients with thin endometrium, who were unresponsive to regular methods (7, 8). Many trials have been designed to assess the therapeutic effects of G-CSF perfusion in patients with thin endometrium; however, concerned with the present findings, the studies have not reached a unanimous conclusion. Some studies suggested that G-CSF infusion increased endometrial thickness significantly, but there was no corresponding improvement in embryo implantation and clinical pregnancy rates (9). Another study suggested that, after G-CSF infusion, a significant increase was detected in endometrium thickness, and there was a significant improvement in embryo implantation and clinical pregnancy rates in women with resistant thin endometrium (10). It is not, yet, definitely known whether intrauterine perfusion of G-CSF can increase rates of embryo implantation and ongoing pregnancy in the *in vitro* fertilization (IVF) population with cycle cancellations due to thin endometrium that is unresponsive to regular therapies or whether there are safer and more effective therapies to improve IVF outcomes in these patients.

As a non-drug and non-invasive therapy, transcutaneous electrical acupoint stimulation (TEAS) has been widely accepted by many infertile women as a complementary treatment in IVF centers in China (11). It has been demonstrated that TEAS exhibits a potential role in improving menstrual regularity and changing electrophysiology of the human endometrium, leading to an enhanced endometrial receptivity (12). Furthermore, acupuncture may positively affect the endometrial morphology and micro-circulation, and decrease muscle motility in the uterine, which facilitate embryo implantation (13). The mechanism of acupuncture improving endometrial receptivity is poorly known. However, it does not affect its widespread use by physicians of traditional Chinese medicine as an adjuvant therapy to achieve better endometrial receptivity (14).

Given the endometrial proliferation of C-GSF and endometrial receptivity improvement of TEAS, the question arises whether G-CSF combined with TEAS may improve embryo implantation and ongoing pregnancy rates in women with unresponsive thin endometrium. The current study is designed to answer this question.

## Methods

### Study Population and Design

Ninety-nine patients with previous IVF cycle cancellations due to unresponsive thin endometrium were enrolled in the present study at the reproductive department of the Second Affiliated Hospital of Chengdu University of T.C.M. from May 2017 to October 2019. This trial was performed with the permission of the hospital ethics committee, and a written informed consent was given to each participant.

Endometrial thickness was measured with transvaginal ultrasound in the longitudinal plane, at the widest section of the endometrial stripe and the myometrium of the central longitudinal axis of the uterus. Thin unresponsive endometrium was defined as one with a thickness <7 mm on the day of triggering ovulation in fresh cycles or one that is resistant to treatment for more than 10 days with the maximum dose of estradiol administered orally and vaginally (10 and 3 mg/day, respectively) and with vaginal sildenafil citrate in hormone replacement treatment (HRT) cycles. The thin unresponsive endometrium referred to in the present study was defined as the thickness of endometrium that would not increase any more after reaching a certain extent (<7 mm), though, at a high blood level of estrogen.

Inclusion criteria were as follows: women aged 20–40 years (a) with a basal FSH concentration □10 IU/L, (b) with at least one cycle cancellation due to thin unresponsive endometrium in IVF treatment, (c) without any other endometrial problems (such as fibroids, submucous myoma, Asherman's syndrome, and polyps) identified by diagnostic hysteroscopy, and (d) with no contraindications for G-CSF intrauterine perfusion (such as sickle cell disease, chronic neutropenia, renal insufficiency, history of malignant tumors, and congenital fructose intolerance). During the trial period, patients with unresponsive thin endometrium were given options as follows: (a) canceling embryo transfer and receiving endometrium treatment in sub-sequent frozen-thawed embryo transfer (FET) cycle and (b) transferring the embryo into an inadequately thick endometrium. About 56 women who met the inclusion criteria and agreed to accept G-CSF and TEAS treatment in subsequent FET cycles were prospectively enrolled. Patients were randomly assigned to the G-CSF perfusion combined with TEAS group and the G-CSF perfusion only group by a computer-generated randomization table. Forty-three women with thin endometrium who underwent FET with HRT protocols, during January 2016 and May 2017, were retrospectively included as controls according to the inclusion criteria (a), (b), and (c). The controls who received special treatment other than estrogen during the period of endometrium preparation were excluded.

### The Endometrial Preparation Program

The endometrium was prepared with HRT protocol in all FET cycles, in which, estradiol and progesterone were administered sequentially to mimic the natural cycles. Estradiol valerate (Bayer, Germany) was taken orally throughout the cycle. The starting dose was 4 mg/day on Day 3, was gradually increased to 10 mg/day, and was maintainedat that level. Endometrial thickness and pattern were monitored with transvaginal ultrasound before each G-CSF perfusion treatment. Transvaginal progesterone gel (Crinone® 8%, Fleet laboratories Limited, England) was started on Day 18 of the cycle, at a dose of 90 mg/day, and continued until the pregnancy test 2 weeks after embryo transfer.

### The Intrauterine Perfusion of G-CSF

Granulocyte colony-stimulating factor was perfused into the uterine cavity on Day 8, 11, 14, and 17 of the cycle (10). The infusion of G-CSF was made under transabdominal ultrasound monitoring with full bladder before transfer. About 300 μg of G-CSF (150 μg/0.9 ml, Qilu Pharmacy Co., Ltd., China) was pulled into a 2 ml syringe, and an embryo transfer catheter was inserted into the uterine cavity. Then, the G-CSF was pushed into the uterine, and the catheter was gently moved back and forth to make the medicine spread evenly on the endometrium. Finally, a small amount of air was drawn into the syringe, and the remaining G-CSF in the catheter was pushed into the uterine cavity. Participants were advised to lie on bed for 30 min after perfusion. Endometrial thickness was evaluated on the day of progesterone administration by the same experienced sonographer.

### The Treatment of TEAS

Transcutaneous electrical acupoint stimulation was performed in the G-CSF combined with TEAS group at acupuncture points SP8 (Diji), PC6 (Neiguan), GV20 (Baihui), ST29 (Guilai), and LR3 (Taichong). The acupoints were determined with reference to the 2nd edition of the Standard Acupuncture Nomenclature (WHO Standard Acupuncture Point Locations in the Western Pacific Region). TEAS was performed by an electro stimulator (EN-Stim 4, Enraf Nonius) through self-adhesive skin electrodes. The disperse-dense wave and 2 Hz in frequency were selected. The intensity range was set at 10–20 mA, depending on the discomfort intolerance. Stimulation reaction (propagate along the corresponding meridians, or numbness, soreness, and distension around the acupoints which termed the “DeQi” sensation) was elicited. Each therapy session lasted for 30 min, during which intensity would be reevaluated every 6 min and reset if necessary. TEAS treatments were administered every other day between Day 3 of the cycle and the day of embryo transfer, equaling a total of six times. The TEAS treatment was operated by two experienced Chinese medicine practitioners in this hospital.

### The Assessment of Embryos and Pregnancy Outcomes

Embryo quality was assessed morphologically by the percentage of cytoplasmic fragmentation, number of blastomeres, and symmetry. Day 3 good embryos were defined as the eight-cell stage with <15% fragmentation. Day 5 good embryos were defined as expanded blastocysts with at least grade B trophectoderm and inner cellmass.

The clinical pregnancy rate was the primary outcome. Clinical pregnancy was defined by the detection of a gestational sac and fetal heart on ultrasound examination at 6–8 weeks of gestation. Ectopic pregnancies were not included as clinical pregnancies. The secondary outcome is endometrium thickness and embryo implantation rate. Embryo implantation rates were evaluated by the number of gestational sacs seen on ultrasound at 7–8 weeks per the number of embryos transferred. Ongoing pregnancy was defined as a pregnancy proceeding beyond 20 weeks gestational stage.

### Statistical Method

All analyses are conducted using SPSS 18.0. Continuous data were presented as mean ± SD and compared by using a *t*-test, an ANOVA, and the Kruskal-Wallis test. The categorical data were compared by using Fisher's exact test and the chi-square test. A *p*-value smaller than 0.05 was considered statistically significant.

## Results

Baseline characteristics of the patients included are displayed in Table 1. In terms of clinical variables, no significant difference was suggested among the three groups in age, indicators of ovarian function, and infertility. One patient in each treatment group and eight in the control group were suggested to cancel FET due to poor endometrium pattern and thickness. The control group presented a significantly higher rate of cycle cancellation than the two treatment groups (3.70, 3.45, and 18.60%; *P* = 0.044).

**Table 1:** Baseline characteristics of the study patients.

	**G-CSF group (*n* = 27)**	**G-CSF with TEAS (*n* = 29)**	**Control group (*n* =43)**	***P***
Age (years)	31.7 ± 5.44	32.98 ± 5.12	32.33 ± 4.47	0.514
BMI (kg/m^2^)	21.37 ± 2.78	21.79 ± 2.31	22.01 ± 3.24	0.472
Baseline FSH (IU/L)	6.14 ± 1.75	6.71 ± 1.98	6.58 ± 2.11	0.591
AMH (ng/ml)	3.97 ± 3.18	4.23 ± 3.77	4.16 ± 3.25	0.218
Type of infertility (%)				0.820
Primary	22.22 (6/27)	17.24 (5/29)	23.26 (10/43)	
Secondary	77.78 (21/27)	82.76 (24/29)	76.74 (33/43)	
Duration of infertility (years)	4.72 ± 1.49	5.26 ± 1.72	4.83 ± 1.19	0.611
Gravidity (*n*)	2.17 ± 1.91	1.95 ± 1.79	2.02 ± 1.98	0.247
Parity (*n*)	0.30 ± 0.61	0.17 ± 0.38	0.23 ± 0.55	0.675
Cycle cancellation (%)	3.70 (1/27)	3.45 (1/29)	18.60 (8/43)	0.044

*BMI, body mass index; FSH, follicle-stimulating hormone; AMH, antimullerian hormone. Values are presented as mean ± SD*.

A comparison of endometrial thickness in patients before and after treatment of TEAS and (or) G-CSF perfusion in FET cycles is documented in Table 2. The baseline endometrium thickness shows no significant difference among the three groups (5.97 ± 0.60, 6.14 ± 0.52, and 6.23 ± 0.46; *P* = 0.126). In the two treatment groups, the endometrium thickness was statistically significantly increased after treatment (5.97 ± 0.60, 7.52 ± 0.56, 6.14 ± 0.52, and 7.66 ± 0.44; *P* = 0.00 and 0.00, respectively). However, in the patients treated with G-CSF perfusion, adjuvant TEAS did not contribute to an increase of the endometrial thickness (1.55 ± 0.52 and 1.52 ± 0.36; *P* = 0.797).

**Table 2:** Comparison of endometrium thickness.

	**G-CSF group (*n* = 27)**	**G-CSF with TEAS (*n* = 29)**	**Control group (*n* = 43)**	***P***
Endometrial thickness (mm) before G-CSF infusion	5.97 ± 0.60^a^	6.14 ± 0.52^b^	6.23 ± 0.46	0.126
Endometrial thickness (mm) after G-CSF infusion	7.52 ± 0.56^a^	7.66 ± 0.44^b^		0.305
Δendometrialthickness (mm)	1.55 ± 0.52	1.52 ± 0.36		0.797

*Values are presented as mean ± SD.*

a *Comparison of endometrial thickness in patients before and after treatment of G-CSF perfusion. p = 0.001 denotes significance.*

b *Comparison of endometrial thickness in patients before and after treatment of G-CSF perfusion and TEAS. p = 0.000 denotes significance*.

There was no significant difference in the number and type of transferred embryos and good embryos among the three groups (*p* = 0.811, 0.910, and 0.523). The G-CSF combined with the TEAS group had a significantly higher embryo implantation rate than the control group (33.33 and 29.41%; *p* = 0.034); however, in patients treated with G-CSF perfusion, TEAS did not achieve a higher implantation rate (33.33 and 29.41%; *p* = 0.412). In addition, the increase in implantation rate in the G-CSF perfusion only group, while nominally greater than the controls, was not significantly different (29.41 and 17.39 %; *p* = 0.091). Comparative analysis of the three groups did not suggest any significant difference in rates of clinical pregnancy, of spontaneous abortion, and of ongoing pregnancy, though, there were nominally higher rates in the G-CSF perfusion combined with TEAS group than the other two groups (Table 3).

**Table 3:** Characteristics of embryos transferred and pregnancy outcomes.

	**G-CSF group (*n* = 26)**	**G-CSF with TEAS (*n* = 28)**	**Control group (*n* = 35)**	***P***
Embryos transferred (*n*)	1.96 ± 0.19	1.93 ± 0.26	1.97 ± 0.30	0.811
Type of embryos				0.910
Day 3 embryos transferred (%)	73.08 (19/26)	67.86 (19/28)	71.42 (25/35)	
Day 5 embryos transferred (%)	26.92 (7/26)	32.14 (9/28)	28.57 (10/35)	
Good embryos transferred (n)	1.81 ± 0.40	1.86 ± 0.36	1.91 ± 0.28	0.523
Embryo implantation (%)	29.41 (15/51)^a^^b^	33.33 (18/54)^a^^c^	17.39 (12/69)^b^^c^	
Clinical pregnancy (%)	46.15 (12/26)	53.57 (15/28)	34.29 (11/35)	0.192
Spontaneous abortion (%)	9.09 (1/12)	7.14 (1/15)	16.67 (2/12)	0.816
Ectopic pregnancy (*n*)	0	0	1	
Ongoing pregnancy (%)	38.46 (11/26)	46.43(14/28)	25.71 (9/35)	0.125

*Values are presented as mean ± SD.*

a *p = 0.412.*

b *p = 0.091.*

c *p = 0.034*.

## Discussion

Adequate endometrial thickness is essential for a successful pregnancy. Endometrium thinner than 7 mm or less has been found to result in a lower pregnancy rate and a higher rate of cycle cancellation and embryo cryopreservation, which leads to a clinical dilemma. Thus, active treatments are recommended in patients with thin endometrium during IVF cycles; however, treatment strategies and regimens proposed are widely considered inefficient (15), so it is still a problem to be solved.

Since 2011, many researchers have shed light on the treatment effects of G-CSF perfusion on unresponsive thin endometrium (9, 16), but none provided a definitive answer. The current research was designed to assess the effect of G-CSF perfusion and TEAS on thin endometrium before FET. We found that G-CSF has a potential role in improving endometrium thickness in patients with thin unresponsive endometrium in FET treatment cycles. In addition, when combined with TEAS, G-CSF perfusion treatment also improves the embryo implantation rate.

These findings are consistent with previously reported data (10), the thickness of endometrium had a significant increase after intrauterine G-CSF perfusion, from 5.97 ± 0.60 and 6.14 ± 0.52 to 7.52 ± 0.56 and 7.66 ± 0.44. It seems improbable that thin endometrium unresponsive to regular treatments would have a sudden and spontaneous proliferation. Therefore, the improvement in endometrial thickness demonstrated, in this research, is, with great likelihood, associated with G-CSF perfusion and (or) TEAS. This result is also very consistent with the study conducted by Gleicher et al. (7), in which, the thickness of endometrium significantly increased in women treated with G-CSF. However, in contrast to the study of Gleicher, patient population in this study was young and had a normal ovary functioning. In addition, the clinical pregnancy rate and the ongoing pregnancy rate did not demonstrate any significant difference between the G-CSF treatment groups and controls. Considering the age and ovarian reserve of the control patient population, with an age of 32.33 ± 4.47 years and an AMH value of 4.16 ± 3.25 ng/ml, and taking the good quality embryos into account, though, with thin endometrium, we obtained a clinical pregnancy rate of 34.29%. The rate was considered to be quite remarkable. In reproductive centers, where the baseline pregnancy rates have already been high, the relatively increased effectiveness of additional procedures, such as G-CSF or acupuncture, may not be detected in a small sample size. That is maybe one potential explanation for these findings.

Although, prospectively randomized studies are necessary to document the effects of G-CSF on thin endometrium before a final judgment, this study alone suggests that G-CSF intrauterine perfusion enhances endometrial proliferation to a statistically significant degree. The result raises a question: How does G-CSF increase endometrial thickness in such a short period? However, little is known about the mechanism. G-CSF is a kind of glycoprotein that has the function of cytokines and growth factors. It is primarily found in circulating monocytes, macrophages, and endothelial cells. In the nervous system, G-CSF not only stimulates nerve growth and regeneration but also has a function of anti-apoptosis; therefore, it has been proposed to apply for neurodegenerative disorders as a potential therapeutic method (17). Tanaka and Umesaki (18) investigated the effects of G-CSF perfusion on differentiation and proliferation in human endometrial stromal cells and concluded that G-CSF enhanced stromal cell decidualization in both paracrine and autocrine ways. In another study, G-CSF was reported to increase the amount of hematopoietic and mesenchymal stem cells in bone marrow (19), which raised the speculation about the stem-cell-like ability of G-CSF in endometrial expansion.

In the two G-CSF perfusion groups, we failed to detect a significant additional effect of TEAS on endometrial proliferation and reproductive outcomes, though, the TEAS group was nominally higher. The result, therefore, suggested that TEAS did not offer any clinical benefit in women treated with G-CSF perfusion; however, this is a conclusion which must be treated with caution: the sample size of the present trial was small because the participants were adversely selected to an unusual degree. Among the 99 patients in this study, some had not been able to reach a minimal endometrium thickness (7 mm) in previous IVF cycles, while some had suffered from several canceled cycles due to thin unresponsive endometrium (?7 mm). Therefore, we cannot conclusively rule out the possibility that the effects of G-CSF perfusion combined with TEAS in a large sample size study may be more noticeable. The noteworthy nominal advantages of TEAS treatment in favor of patients with G-CSF intrauterine perfusion may point toward that possibility. As noted above, whether G-CSF combined with TEAS, indeed, improves endometrium proliferation and pregnancy outcomes compared with G-CSF treatment only remains, however, to be determined and is subject to further studies.

Reproductive outcomes are the main indicators for clinicians to assess the therapeutic effects of G-CSF perfusion; however, we failed to obtain a higher implantation rate in the G-CSF perfusion-only group compared with controls. Therefore, we could speculate that an increase in endometrial thickness may not be the only factor contributing to a higher embryo implantation rate. The previous study has confirmed that probably 60% of implantation failures in IVF cycles are attributed to poor endometrial receptivity (20). Therefore, good endometrial receptivity holds the key to improving reproductive outcomes. TEAS is a safe, mild, and non-invasive treatment with relatively fewer side effects. As an adjuvant therapy with high patient acceptance, acupuncture helps improve the reproductive outcomes of patients with IVF treatment (14). A previous study (21) has documented that acupuncture significantly improves endometrial and sub endometrial microcirculation, uterine artery blood flow, and spiral artery blood flow, which are associated with endometrium acceptability. The above discussion provides a reasonable explanation for the result in the current research, which suggested a statistically significantly higher implantation rate in G-CSF with the TEAS group.

There are also some limitations in the current study. First, the sample size (but a highly selected patient population) is not large enough and the control is retrospective, which is primarily due to the low morbidity rate of thin resistant endometrium and strict inclusion criteria. It is suggested that it is not practical to recruit a large patient population from one center in about 2 years. Due to the selection bias based on the inclusion method of the patient, the results of the study should be interpreted cautiously. Second, most of the included patients were at a young age with normal ovarian function. Therefore, the conclusions may not apply to all women with thin endometrium, especially to older women than those investigated in this study, and cautions should be taken in over-interpreting these results.

## Conclusion

Granulocyte colony-stimulating factor intrauterine perfusion can significantly improve endometrium thickness in women with thin unresponsive endometrium in FET cycles. The present study does not suggest a beneficial influence from G-CSF perfusion on pregnancy outcomes; however, when combined with TEAS, G-CSF perfusion has a positive effect on embryo implantation. In addition, randomized controlled trials are highly demanded to provide high-grade evidence regarding the clinical pregnancy rate after G-CSF perfusion treatment.

## Data Availability Statement

The raw data supporting the conclusions of this article will be made available by the authors, without undue reservation.

## Ethics Statement

The studies involving human participants were reviewed and approved by the hospital ethics of Reproductive & Women-Children Hospital, Chengdu University of Traditional Chinese Medicine. The patients/participants provided their written informed consent to participate in this study.

## Author Contributions

LS designed the research and wrote the manuscript. XS and QZ conducted the fieldwork and analyzed data. SZ revised the manuscript. All authors read and approved the final manuscript.

## Conflict of Interest

The authors declare that the research was conducted in the absence of any commercial or financial relationships that could be construed as a potential conflict of interest.
